# Self‐efficacy in the context of nursing education and transition to practice as a registered practitioner: A systematic review

**DOI:** 10.1002/nop2.1931

**Published:** 2023-07-08

**Authors:** Mousa Abusubhiah, Nuala Walshe, Rena Creedon, Brendan Noonan, Josephine Hegarty

**Affiliations:** ^1^ School of Nursing and Midwifery University College Cork Cork Ireland

## Abstract

**Aim:**

The aim of this systematic review is to identify, describe and synthesize evidence from experimental studies conducted to measure and conceptualize self‐efficacy within the context of nursing education and the transition of nursing students to practice as a registered practitioners.

**Design:**

Systematic review.

**Methods:**

Papers were screened by four independent reviewers, and data were extracted using a standardized data extraction tool. The Preferred Reporting Items for Systematic Reviews and Meta‐Analyses (PRISMA) guidance and checklists were used to guide this review.

**Results:**

The review included 47 studies, using a quasi‐experimental pre‐test–post‐test design (*n* = 39) and randomized control trials (*n* = 8). Various teaching and learning interventions were used to enhance self‐efficacy; however, there is no definitive conclusion to be drawn regarding the most effective educational interventions. Various instruments were used in the studies to measure self‐efficacy. 10 of these were related to general self‐efficacy, while 37 instruments measured self‐efficacy in the context of specific skills.

## 1 INTRODUCTION

Nursing education is the bedrock of good nursing practice across the globe (Kraft et al., 2017). Education varies from apprenticeship models to degree level for undergraduate education, with graduate entry programmes emerging globally (Spitzer & Perrenoud, 2006). Governing bodies and regulators vary in terms of educational requirements and standards, programme hours and graduate competencies required in programmes preparing nurses for registration (Baker et al., 2021). Postgraduate education is increasing in importance as more specialist roles develop (Liu et al., 2020). However, it is vitally important for nursing students to accurately and effectively transfer knowledge into practice (Von Colln‐Appling & Giuliano, 2017). One of the theories, which can be used to assess situation‐specific self‐confidence underlining the initiation of certain behaviours which are required for competent performance is Bandura's self‐efficacy theory. Self‐efficacy is defined as one's belief in the ability to perform the desired functions (Bandura, 1994). Many researchers have noted the correlation between self‐efficacy and academic performance (Rogers, 2018) and the performance of certain tasks (Bandura, 1977; Honicke & Broadbent, 2016). Nursing students face challenges in transitioning into clinical practice and working as registered practitioners and registered practitioners face challenges in responding to their changing roles. It has been suggested that self‐efficacy has an important role in the delivery of quality nursing care, supporting nurses in developing their professional identity and enhancing their performance and development of their competence (Kim & Sim, 2020; Yao et al., 2021; Yu et al., 2021). For this reason, educators and researchers globally have considered self‐efficacy as one of the target process level and terminal programme outcomes which can influence nursing practice.

Self‐efficacy is a vital component for independent performance in nursing. Bandura has described self‐efficacy as one's belief in the ability to perform the desired functions in their role (Bandura, 1995). Self‐efficacy is an important motivator for medical and nursing students' development (Klassen & Klassen, 2018) and is key to nursing students' and clinical nurses' performance in their roles (Mohmmdirezi et al., 2015). Therefore, developing and implementing education programmes which facilitate the development of self‐efficacy is essential for healthcare professionals. Over the past 20 years, self‐efficacy has been linked to academic achievement and workplace performance (Artino, 2012; Goldshmidt, 2018).

Although the effectiveness of educational programmes is well documented, specific analysis of self‐efficacy as an outcome is less evident. For example, based on a systematic review of 19 included articles, McCutcheon et al. (2015) found limited evidence in support of using blended learning approaches to teach clinical skills in nursing education programmes. Only two papers reported on self‐efficacy, and they all used different outcome measures. McMullan et al. (2011) found no significant differences in students' self‐efficacy before and after drug calculation training. McConville and Lane (2006), using an online video increased students' self‐efficacy scores when faced with difficult situations. Furthermore, a number of authors conducted systematic literature reviews of self‐efficacy with varying foci: concluding that high levels of academic self‐efficacy significantly increased university students' academic performance (Honicke & Broadbent, 2016); self‐efficacy is important for healthcare professionals' communication skills, where training in these skills can enhance their performance and self‐efficacy (Mata et al., 2021); considering a breastfeeding education programme during pregnancy and continued to the first week of postpartum. Self‐efficacy provides an important opportunity for health professionals to improve breastfeeding confidence among mothers when they encounter breastfeeding problems (Maleki et al., 2021).

Systematic reviews, by synthesizing the available evidence, enable the consistency of results to be analysed and provide a more solid basis for decision‐making. Several systematic reviews exploring the role of self‐efficacy in nursing studies have been published. However, little attention has been given to understanding self‐efficacy baseline levels and the choice of pedagogy or the most effective teaching methodologies to support the development of self‐efficacy of Registered Nurses and nursing students that would allow us to pool the different results. Therefore, considering self‐efficacy's importance, an in‐depth systematic review of the literature will be presented in this paper, which will explore the impact of educational interventions on nurses' self‐efficacy.

## 2 THE REVIEW

### 2.1 Aim

The aim of this systematic review is to identify, describe and synthesize evidence from experimental studies conducted to measure and conceptualize self‐efficacy within the context of nursing education and the transition of nursing students to practice as a registered practitioner. A systematic review of empirical literature will be used to broaden the conceptualization and understanding of the evidence, due to the inclusive nature and the predefined rigorous methodological processes used. Specifically, in this review, we sought to answer the following questions:

- How do education programmes impact the self‐efficacy of nursing students and Registered Nurses?
- What educational programme characteristics have been linked with changes in self‐efficacy?
- What role does self‐efficacy have in facilitating the transition of nursing students from an educational programme to practice as a registered practitioner?

### 2.2 Design

A systematic literature review design was selected to synthesize empirical study findings and to address the research questions. The Preferred Reporting System for Systematic Reviews and Meta‐Analyses (PRISMA) guidance and checklist was followed in this study (Page et al., 2021).

### 2.3 Search methods

Inclusion and exclusion criteria were drafted based on review questions and reported in accordance with the PICOS framework (Population, Interventions, Comparators, Outcomes and Setting/Study design) (Santos et al., 2007; Table 1). The review considered experimental studies published from 01 January 2010 to 31 March 2021. Papers were included if: (a) they focused primarily on nursing students or Registered Nurses undertaking any educational programme (undergraduate or graduate entry or postgraduate or continuing professional development (CPD) programmes); (b) measured or explored self‐efficacy as an outcome (c) reported on studies conducted in healthcare settings or educational institutions.

**TABLE 1:** Overview of inclusion and exclusion criteria using the PICOS model.

PICOS	Inclusion criteria	Exclusion criteria
Population	Nursing students and registered nurses undertaking any educational programme (undergraduate or graduate entry or postgraduate or CPD programmes) Interprofessional learning, including nursing students or registered nurses where the data pertaining to nursing students and nurses can be clearly extracted	Other healthcare professionals, dental nurses, veterinary nurses, health care assistants and doctors
Interventions, Variable of Interest	Experimental studies which sought to understand the impact of a specific educational programme/intervention where self‐efficacy was explored as an outcome variable	Patient educational programmes Measurement of self‐efficacy in the context of correlational or interventional studies relating to general concepts such as nursing/midwifery's personal self‐image, eating habits, tobacco cessation and engagement in exercise Non‐interventional studies
Comparison	As reported in the study	
Outcome of interest	Research reporting on self‐efficacy as an outcome variable Self‐efficacy is mentioned in the title or abstract	Related concepts, for example confidence where self‐efficacy is not the focus Studies that present the psychometric evaluation of an instrument Studies with no self‐efficacy outcome data Writing self‐efficacy
Studies	All experimental designs Publications in peer‐reviewed journals	Non‐interventional empirical designs (i.e. qualitative, quantitative and mixed methods design) Thesis, conference abstracts, opinion pieces, study protocol, literature reviews using other designs, systematic review
Limits applied	Articles published 1 January 2010–1 March 2021 English language only	

The search strategy was developed iteratively and reviewed by five authors. In a preliminary scoping search of databases, we tested and refined the search strategy as a group, which ensured the sensitivity of the search in sourcing key papers within the search results. A comprehensive search was conducted using the EBSCO host platform and key databases, that is Educational Resources Information Center (ERIC), the Cumulative Index to Nursing and Allied Health Literature (CINAHL) and Medline. A title or abstract keyword search was conducted using Boolean terms (see Table 2).

**TABLE 2:** Search strategy outlined using PICO.

	Search modes—Boolean/Phrase Interface—EBSCOhost Research Databases ERIC, CINAHL plus with full text and Medline	Result	Explanation
S1	TI ((nurse OR nursing OR midwifery OR midwife)) OR AB ((nurse OR nursing OR midwifery OR midwife))	83,244	These terms were chosen to target the population
S2	TI ((educat* OR program*OR train* OR graduat* OR clinical OR competen* OR student OR undergraduate OR preregistration OR "pre‐registration" OR baccalaureate OR prelicensure OR "pre‐licensure")) OR AB ((educat* OR program* OR train* OR graduat* OR clinical OR competen* OR student OR undergraduate OR preregistration OR "pre‐registration" OR baccalaureate OR prelicensure OR “pre‐licensure"))	979,778	These terms were chosen to target the education programmes' context
S3	TI ((self‐efficacy OR “self efficacy”)) OR AB ((self‐efficacy OR “self efficacy”))	16,553	These terms were chosen to target the main concept
S4	(S1 AND S2 AND S3)	723	These terms were chosen to target the empirical literature in a nursing education context involving self‐efficacy as a major concept or variable

The papers identified by the electronic database search were imported into a Mendeley library at https://www.mendeley.com, and duplicates were removed. The remaining citations were transferred to Rayyan, an online screening tool (Ouzzani et al., 2016). Papers were blindly screened on the basis of title and abstract, and irrelevant records were excluded. Following that, papers that might be eligible were assessed in full text. Title, abstract and full‐text screening were conducted independently by two reviewers based on predefined inclusion and exclusion criteria. Any disagreements at the full‐text screening were resolved through discussion and consensus.

### 2.4 Quality assessment

Five authors in pairs independently assessed quality and scored included papers using the Mixed Methods Appraisal Tool (MMAT; Hong et al., 2018; see Appendix S1). The tool facilitated the assessment of included studies based on their design category. Studies that met the following criteria were rated as follows: 0% for one criterion met, 25% for two, 50% for three, 75% for four or 100% for five criteria met.

### 2.5 Data extraction

A standardized data extraction template was used to extract the data (see Appendix S2). The data from the identified studies were extracted by one reviewer, and the accuracy of the extraction process was verified by another reviewer. Disagreements between reviewers were resolved by consensus or by involving a third reviewer.

The data extracted included specific details about the author(s) and year, country and settings; aim(s); study design; study population; exposure/intervention description; outcome variables; self‐efficacy theory and definition, self‐efficacy data collection method and instrument; and the self‐efficacy results. The findings are organized based on review questions as follows: (a) what is the impact of education programmes on the self‐efficacy of Registered Nurses (RNs) and/or nursing students? (b) what educational programme characteristics have been linked to changes in self‐efficacy? and (c) what role does self‐efficacy play in facilitating the transition of nursing students from an educational programme to practice as a registered practitioner?

### 2.6 Data synthesis

Researchers intended to pool quantitative study results and conduct a statistical meta‐analysis. According to Field and Gillett (2010), this approach was not considered valid or feasible due to the considerable heterogeneity of interventions and outcome measures in included the studies. Therefore, the findings are summarized as a narrative without meta‐analysis with supporting tables (Popay et al., 2006; Ryan, 2013). Where effect sizes were not reported by study authors, the standard effect sizes for the primary outcome (changes in self‐efficacy) were calculated using an online calculator (https://www.uccs.edu/lbecker/). Cohen's *d* statistic was used to report the effect size *r* (a Pearson correlation coefficient). As *d* is the standardized mean difference between two groups; *r* is the proportion of variance that can be explained (between zero and one). In the case where means and standard deviations were not fully reported, they were computed using other data, such as *t*‐value and df methods.

The *r* statistic is generally interpreted as having a small effect of 0.10, a medium effect of 0.30 and above and a large effect of 0.50 and above (Anon, 2021). In this study, *r* was considered negligible when it was <0.2, so that no change was seen in mean self‐efficacy scores; this indicates that the means of two groups differ by <0.2 standard deviations and that the difference is extremely small or negligible, even when statistically significant (Polit & Tatano‐Beck, 2012).

## 3 RESULTS

### 3.1 Selection of the studies

**FIGURE 1:**
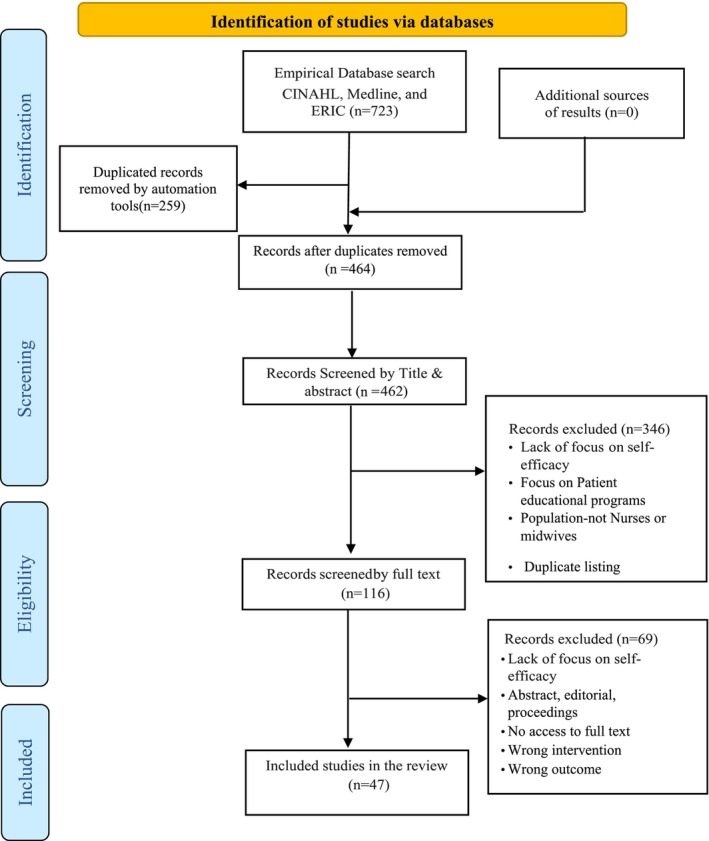
This diagram illustrates the empirical search strategy. Adapted from: Page et al. (2021).

The search of the databases revealed 723 citations that were exported to Mendeley. A total of 259 duplicates were removed from Mendeley; 464 were imported to Rayyan, where two duplicates were removed. Finally, a total of 462 citations were screened at the initial title and abstract screening stage, and 346 irrelevant records were excluded from the results (Figure 1).

The full text of the remaining 116 papers was screened, and 69 papers that did not meet the review eligibility criteria were excluded, resulting in 47 studies that met the inclusion criteria. In addition to the included articles, the references of the included articles were searched manually, and no additional papers were found. The study identification, screening and selection process are presented in Figure 1.

### 3.2 Study characteristics

A total of 47 studies were included in accordance with the inclusion and exclusion criteria. Most of the studies were done in America (*n* = 21); other studies took place in South Korea (*n* = 6), Turkey (*n* = 3), Iran (*n* = 5), Canada (*n* = 2), Taiwan (*n* = 2), Netherlands, Australia, Jordan, Saudi Arabia, Denmark, Egypt, Thailand and Mexico (*n* = 1 each) (Table 3).

**TABLE 3:** Key study characteristics (*N* = 47).

Characteristic	Details
Country	USA (*n* = 21) South Korea (*n* = 6) Turkey (*n* = 3) Iran (*n* = 5) Canada (*n* = 2) Australia (*n* = 1) Netherlands (*n* = 1) Taiwan (*n* = 2) Jordan (*n* = 1) Saudi Arabia (*n* = 1) Denmark (*n* = 1) Egypt (*n* = 1) Thailand (*n* = 1) Mexico (*n* = 1)
Setting	University and academic settings (*n* = 26) Community healthcare centres (*n* = 6) Hospital and acute care settings (*n* = 15)
Population	Undergraduate nursing students (*n* = 3204) Postgraduate nursing students (*n* = 2112) Registered Nurses (*n* = 896) Nurses and allied health care professionals (*n* = 1463) of which nurses (*n* = 622)
Study design	Quasi‐experimental pre‐test–post‐test design (*n* = 39) Randomized control trial RCT (*n* = 8)
Theoretical underpinning relating to self‐efficacy	Self‐efficacy theory (Bandura, 1977, 1986, 1995, and 2006) (*n* = 6) Social cognitive theory (Bandura, 1986) (*n* = 5)
Theoretical underpinning relating to educational/psychological educational interventions	Experiential learning theory (Kolb, 1984) (*n* = 4) Theory of planned behaviour (Ajzen, 1991) (*n* = 1) Structural empowerment theory (Kanter, 1993) (*n* = 1) Accelerated implementation methodology framework (Centre for Health care redesign, 2014) (*n* = 1) Nursing model of skill acquisition (Benner, 2004) (*n* = 1) Simulation framework (Jeffries, 2005) (*n* = 1) Personal reflection theory (Kenney, 2006) (*n* = 1)
Sample size (min–max)	6–644

The study design included a quasi‐experimental pre‐test–post‐test design (*n* = 39) and randomized control trial designs (*n* = 8) (Table 3). Sample sizes ranged from 6 (Crocetti, 2014) to 644 participants (Nørgaard et al., 2013). The included studies had a total of 6834 participants, including undergraduate nursing students (*n* = 3204), postgraduate nursing students (*n* = 2112), Registered Nurses (*n* = 896) and nurses and allied healthcare professionals (*n* = 1463) of which 622 were nurses.

Various educational modalities and programmes were highlighted in the reviewed literature (Appendix S3). Intervention types included single learning modalities like online training modules (*n* = 4), simulation learning (*n* = 4), multimodality learning programmes (multimodality‐based simulation) (*n* = 10) and blended learning (*n* = 10). Other traditional educational programmes included didactic learning approaches (*n* = 21).

### 3.3 Quality assessment

There were 47 studies that met all five Mixed Methods Appraisal Tool assessment criteria, indicating the studies' quality and methodology being generally acceptable to answer research questions. (Appendix S1). The quality was highest in quasi‐experimental pre‐test–post‐test design studies, while the randomized control trial studies scores were lower. Many studies suffered from weaknesses with respect to sampling strategies, representativeness of samples, attrition rates and consideration of possible confounders in their design or analysis. For example, baseline data was not comparable between groups. However, there were strengths in the method of measuring the outcome data and reporting complete outcome data. There were no studies that scored 0 and 4 (quasi‐experimental pre‐test–post‐test design studies) met all the criteria relevant to their study type (Choi & Park, 2017; Imus et al., 2017; Larsen & Reif, 2011; Moore et al., 2019).

### 3.4 Theoretical perspectives around self‐efficacy

Of the reviewed studies, 11 were underpinned by theoretical perspectives relating to self‐efficacy theories. In addition, studies utilized a selection of educational theories, frameworks and models to understand and explain human behaviour and guide the intervention's identification, development and implementation (Table 3). The most used definition or explanation of self‐efficacy within the review referenced Bandura's work (Bandura, 1977, 1986, 1995, 2006), defining that self‐efficacy is ‘the individual's judgment of their capabilities to organize and execute the necessary action required to achieve a designated level of performance’ (Cino et al., 2018; Darkwah, 2011; Doucet & Rhéaume, 2020; Hwang & Kim, 2020; Imus et al., 2017; Larsen & Reif, 2011; Lee et al., 2019; Said et al., 2021; Saied, 2017; Saxton, 2012) (*n* = 10).

### 3.5 Measurement of self‐efficacy

There was significant heterogeneity in the tools (*n* = 47 individual tools) used to measure self‐efficacy within the included studies (see Appendix S4). These measures included short scales that were composed of eight items or less such as the modified self‐learning efficacy scale (Chang et al., 2020; *n* = 8 items), self‐efficacy in pain management scale (Parvizy et al., 2020; *n* = 6 items), academic self‐efficacy scale (Fertelli & Tuncay, 2020; *n* = 7 items) and self‐efficacy regarding sexual media literacy education scale (Baek et al., 2019; *n* = 5 items). The lengthiest scale was the self‐efficacy scale (SES), with 138 items based on Bandura's (1977) writings and a revised version used for the PRONTO pilot study (Walker et al., 2014). All other measures were moderate in length, with 10–45 items.

Within the 47 studies reviewed, self‐efficacy was operationalized as both a global measure and a caring domain/task‐specific measure of self‐efficacy (see Appendix S4). The general self‐efficacy scale (GSES; *n* = 10) was the most popular global measure. In this self‐efficacy scale, 10 items were used with a 4‐point Likert scale, with one as no confidence, four as fully confident, and higher scores indicated higher self‐efficacy (Schwarzer & Jerusalem, 1979, 1993, 1995, and 2010, as cited in Saied, 2017; Imus et al., 2017; Rambod et al., 2018; Topbaş et al., 2019; Hwang & Kim, 2020).

Measures specific to caring domains/tasks were used in seven studies. These included the interprofessional teamwork scale (IPT; *n* = 3), self‐efficacy of clinical performance scale (SECP; *n* = 2) and self‐efficacy in evidence‐based practice scale (SE‐EBP; *n* = 2). In the remaining studies, scales adapted from various sources were used (*n* = 1 each). A more recent self‐efficacy tool assessed nurses' self‐efficacy regarding the management of eclampsia with 16 statements and a 5‐point Likert scale to measure nurses' self‐efficacy. Higher scores indicate a higher level of self‐efficacy (Said et al., 2021). Across all studies, the reliability of these measures ranged from Cronbach's *α* = 0.70 (Thompson, 2016) to 0.98 (Iannuzzi et al., 2019). However, evidence within the publications of test–retest reliability and validity was lacking (see Appendix S4).

With regard to scale development, 11 measures were initially published in English and adapted into other languages, the general self‐efficacy scale (GSES) (Turkish *n* = 1; Korean *n* = 2; German *n* = 1), self‐efficacy in evidence‐based practice (SE‐EBP) scale (Mandarin *n* = 1), self‐efficacy in interprofessional collaboration scale (Danish *n* = 1), work efficiency questionnaire‐II (CWEQ‐II) (French *n* = 1), the perceived palliative care self‐efficacy questionnaire (French *n* = 1), the PNKAS self‐efficacy questionnaires in pain management (Persian *n* = 2) and self‐efficacy scale relating to the nurses' management of eclampsia (Arabic *n* = 1).

### 3.6 Self‐efficacy outcomes for single‐group experimental study designs

Among 28 single‐group studies that reported self‐efficacy comparisons, 25 studies showed significant changes in general or subscale self‐efficacy after the intervention (Table 4). Mean self‐efficacy scores improved post‐intervention significantly in 21 of 28 studies. There were only four studies with single‐group data reporting positive changes in self‐efficacy with an effect size *r* ≥ 0.5 (Chang & Levin, 2014; Dunn et al., 2014; Patton, 2018; Walker et al., 2014) (Table 4). Although single‐group studies indicated that interventions increased self‐efficacy, effect sizes for changes in self‐efficacy subscales or overall self‐efficacy ranged from *r* = −0.38 to 0.93. In four studies, the self‐efficacy intervention effects were lower than 0.20, while other studies reported no significant change in self‐efficacy or no data (*n* = 2).

**TABLE 4:** Self‐efficacy outcomes in single‐group studies.

Study first author	Sample	Instrument (s)	Experimental group		Statistically significant change in self‐efficacy*	Outcome
	*N*	Variable (s)				
			Pre‐test mean (standard deviation)	Post‐test mean (standard deviation)	Yes/No	Effect size (*r*)
Ancel (2016)	26	*Instrument*: Self‐Efficacy Scale				
		*Variables*: Self‐efficacy	85.23 (11.8)	94.35 (9.3)	Yes	0.39
Chang (2014)	60	*Instrument*: Self‐ Efficacy in Evidence‐Based Practice tool				
		*Variables*: Self‐efficacy subscale ‐ Finding the evidence ‐ Appraising the evidence	5.97 (1.67) 4.89 (1.99)	8.18 (1.39) 7.38 (1.74)	Yes Yes	0.58 0.55
Choi (2017)	21 (*n* = 13 nurses)	*Instrument*: Adapted Self‐Efficacy Scale				
		*Variables*: Self‐efficacy	29.62 (3.38)	32.90 (2.17)	Yes	0.50
Cino (2018)	75 (*n* = 37 nursing students)	*Instrument*: Interprofessional Learning (SEIL) scale				
		*Variables*: Self‐efficacy for interprofessional education	No scale data	No scale data	Yes	NCC
Crocetti (2014)	6	*Instrument*: The Self‐Efficacy Toward Teaching Inventory (SETTI) tool				
		*Variables*: ‐ Self‐efficacy with teaching strategies ‐ Confidence in assisting students	26.17 52.33	31.17 67.33	NDR NDR	NCC NCC
Curtis (2016)	59	*Instrument*: Self‐Efficacy Questionnaire				
		*Variables*: Self‐Efficacy	Median (IQR): 123.5 (102.75–133)	Median (IQR): 129 (107.5–137.25)	Yes	NCC
Darkwah (2011)	58 Independent samples: 1st years (*n* = 26), 3rd years (*n* = 36)	*Instrument*: The original Health Promotion Disease Prevention Inventory (HPDPI‐M) Variable: ‐ Confidence in knowledge about smoking ‐ Confidence in counselling about smoking	NDR NDR	3.01 (0.53) third year students 2.59 (0.47) first year students 2.79 (0.54) third year students 2.38 (0.59) first year students	Yes Yes	NCC NCC
Dehghani (2020)	40	*Instrument*: The Perceived Palliative Care Self‐efficacy Questionnaire *Variables*: Self‐efficacy	27.7 (7.9)	39.4 (6.9)	Yes	0.62
Doucet (2020)	19	*Instrument*: Work Efficiency Questionnaire‐II (CWEQ‐II) *Variables*: Self‐efficacy	76.58 (13.25)	80.74 (9.11)	No	0.15
Dunn (2014)	26	*Instrument*: Nursing Student Self‐Efficacy Scale (NSSES)				
		*Variables*: ‐ Self‐efficacy of nurse patient communication ‐ Nurse efficacy for physical patient care	4.08 (0.10) 1.81 (0.17)	4.44 (1.49) 2.64 (0.16)	Yes Yes	0.17 0.93
Hagemeier (2014)	192 (*n* = 36 nurses)	*Instrument*: Researcher developed survey self‐efficacy beliefs				
		*Variables*: Self‐efficacy	NDR	NDR		NCC
Hwang (2020)	87	*Instrument*: General Self‐Efficacy Scale (GSE)				
		*Variables*: Self‐efficacy	27.22 (2.60)	28.94 (3.01)	Yes	0.29
Kameg (2010)	38	*Instrument*: Self‐efficacy (General) Scale Self‐efficacy of communications skills scale (VAS)				
		*Variables*: VAS SE Total VAS SE group 1 VAS SE group 2	48.5 50.90 45.71	59.2 62.40 55.20	Yes Yes Yes	NCC NCC NCC
Karlin (2017)	18 (*n* = 7 nurses)	*Instrument*: The Dementia Information, Self‐Care, and Communication (DISC) Scale				
		*Variables*: Self‐efficacy subscale Workshop 1 Workshop 2	25.6 (5.2) 33.0 (7.7)	28.8 (3.8) 45.0 (5.7)	Yes Yes	NCC NCC
Kim (2018)	64	*Instrument*: General self‐efficacy instrument				
		*Variables*: ‐ General self‐efficacy tool ‐ Academic self‐efficacy tool ‐ Social self‐efficacy tool	3.75 (0.52) 3.66 (0.51) 3.87 (0.67)	4.01 (0.50) 3.79 (0.59) 4.16 (0.74)	Yes Yes Yes	0.25 0.12 0.20
Kool (2014)	178 (*n* = 90 nurses)	*Instrument*: Self‐Efficacy in Dealing with Self‐Harm Questionnaire (SEDSHQ)				
		*Variables*: Self‐efficacy	92.59 (16.61)	101.77 (15.73)	Yes	0.27
Lee (2019)	27	*Instrument*: General self‐efficacy scale				
		*Variables*: Self‐efficacy	T0 39.33 (8.49)	T1 42.89 (5.29) T2 46.80 (5.95)	Yes Yes	0.24 0.45
Leithead III (2019)	152 (*n* = 82 nurses)	*Instrument*: The interprofessional teamwork (IPT) scale				
		*Variables*: interprofessional teamwork self‐efficacy ‐ SE Undergraduate Nursing ‐ SE Nurse Anaesthesia students	4.02 4.72	4.82 4.89	Yes No	NCC NCC
Moore (2019)	126 (*n* = 102 MSN, *n* = 24 DNP)	*Instrument*: EBP instrument, self‐efficacy subscale				
		*Variables*: MSN and DNP students' Self‐efficacy MSN student's self‐efficacy	3.40 2.06	4.04 2.52	Yes Yes	NCC NCC
Patton (2018)	63	*Instrument*: Researcher developed self‐efficacy survey				
		*Variables*: Self‐efficacy	59.28 (18.29)	92.47 (8.34)	Yes	0.76
Rambod (2018)	112	*Instrument*: General self‐efficacy (GSE) scale				
		*Variables*: Self‐efficacy	NDR	30.69 (5.88)	Yes	NCC
Said (2021)	40	*Instrument*: Self‐efficacy scale *Variables*: ‐ Practice ‐ Knowledge ‐ Self‐efficacy	T0 No data	T1 immediately post‐intervention, T2 8 weeks post No data	Yes (positive improvement) on T1 and T2	NCC
Saied (2017)	158	*Instrument*: General Self‐efficacy scale (GSE)				
		*Variables*: Self‐efficacy	34.41 (4.45)	30.30 (5.45)	Yes	−0.38
Saxton (2012)	17	*Instrument*: The Self‐Efficacy to Address Disruptive Behaviour Scale (SADBS) *Variables*: Self‐efficacy	NDR	Total mean score pre‐test to post‐test 1, *p* < 0.05 Total mean score pre‐test to post‐test 2, *p* < 0.05	Yes Yes	NCC NCC
Schwindt (2019)	36 (*n* = 13 nurses)	*Instrument*: Self‐efficacy for various aspects of tobacco cessation counselling for patients with mental illness scale				
		*Variables*: Self‐efficacy	5.4	8.0	Yes	NCC
Thompson (2016)	40	*Instrument*: General Self‐Efficacy Scale				
		*Variables*: Self‐efficacy	29.6 (3.6)	32.1 (3.7)	Yes	0.32
Walker (2014)	305 (*n* = 140 nurses)	*Instrument*: Self‐efficacy scale				
		*Variables*: Self‐efficacy in ‐ Obstetric haemorrhage ‐ Shoulder dystocia (Module II) ‐Preeclampsia/Eclampsia (Module II) ‐ Neonatal Resuscitation ‐ General Obstetric Emergency; Module I ‐ General Obstetric Emergency; Module II	68.1 (20.5) 62.0 (23.5) 81.5 (13.6) 78.6 (17.0) 81.6 (15.5) 86 (11.2)	86.5 (11.9) 88.4 (12.2) 92.0 (8.9) 92.1 (8.7) 92.7 (8.0) 92.5 (9.0)	Yes Yes Yes Yes Yes Yes	0.48 0.58 0.42 0.45 0.41 0.30

*Note*: T0 time 0 (pre‐test), T1 time 1 (post‐test), T2 time 2 (delayed post‐test); T3 time 3 (delayed post‐test); T4 time 4 (delayed post‐test).

Abbreviations: CG, control group; EG, experimental group; NA, not available; SE, self‐efficacy; RN, Registered Nurse; NCC, no calculation completed; NDR, no scale data for nursing students reported.

* *p*‐Values reviewed in original studies to ascertain significance.

However, only one of the included pre‐test–post‐test studies (Doucet & Rhéaume, 2020) reported an effect size value for self‐efficacy (*r* = 0.15). As a result, the research team calculated the effect size for each educational intervention to allow for cross‐study comparisons. The standard effect sizes for the primary outcome (changes in self‐efficacy) were calculated using an online calculator (https://www.uccs.edu/lbecker/) where means and standard deviations were fully reported, they were computed using other data, such as *t*‐value and df methods. Moreover, if none of these data were available for computing the effect size, no calculations were completed (NCC) written under the effect size.

### 3.7 Self‐efficacy outcomes for random control and non‐random control two group studies

There were 16 studies that compared two groups. A total of eight studies randomly assigned participants to intervention or control groups; the other eight were non‐randomized two‐group studies (Table 5). 10 of the 16 studies reported significant increases in self‐efficacy from the pre‐test to the post‐test (*p* ≤ 0.05). These studies had an effect size greater than *r* = 0.2 (RCT = 5; non‐RCT = 5). Two studies among RCTs had intervention effects on self‐efficacy that were less than *r* = 0.20. Only seven of the studies reported positive changes in self‐efficacy with a large effect size *r* ≥ 0.5; (RCT = 4), (Ataee et al., 2019; Iannuzzi et al., 2019; Moeini et al., 2019; Phuangngoenmak et al., 2019); (non‐RCT = 3) (Baek et al., 2019; Chu et al., 2019; Sharour, 2019) (Table 5). All two‐group studies included in this review did not report effect size values. Therefore, the standard effect sizes for the primary outcome (self‐efficacy level change) were calculated by the research team.

**TABLE 5:** Self‐efficacy Outcomes for random control and non‐random control two‐group studies.

Study first author	Sample	Instrument (s)	Experimental group		Control group		Statistically significant change in self‐efficacy	Outcome
RCT/non‐RCT‐two group	*N*	Variable (s)	Pre‐test	Post‐test	Pre‐test	Post‐test	Yes/No	Effect size (*r*)
			Mean (SD)	Mean (SD)	Mean (SD)	Mean (SD)		
Ataee et al. (2019) RCT	37: EG 19; CG 18	*Instrument*: Self‐efficacy in clinical performance (SECP) questionnaire *Variables*: Self‐efficacy	129.89 (18.98)	161.26 (6.07)	125.88 (10.15)	119.72 (10.13)	EG and CG post: Yes	EG: T0‐T1: 0.74 CG: T0‐T1: −0.3
Alfes (2015) RCT	77 MN (*n* = 31; BSN (*n* = 46)	*Instrument*: Mental Health Nursing Clinical Confidence Scale *Variables*: Self‐efficacy	No SD	No SD	No SD	No SD	MN No BSN Yes	NCC
Chang et al. (2020) (Non‐RCT‐two groups)	36: EG (*n* = 18); CG (*n* = 18)	*Instrument*: A modified self‐learning efficacy scale *Variables*: Self‐efficacy	NDR	3.68 (0.62)	NDR	3.13 (0.34)	EG: Yes CG: Yes	NCC
Fertelli (2020) (Non‐RCT‐two groups)	68: EG (*n* = 34); CG (*n* = 34)	*Instrument*: Academic Self‐Efficacy Scale *Variables*: Self‐efficacy	19.02 (2.83)	21.65 (3.41)	20.08 (2.53)	20.21 (2.45)	EG & CG Yes	EG: 0.39 CG: 0.12
Chu (2019) (Non‐RCT‐two groups)	151; EG (*n* = 76; CG (*n* = 75)	*Instrument*: Self‐efficacy in evidence‐based practice (SE‐EBP) scale *Variables*: Self‐efficacy	T0 62.76 (21.66)	T1 89.03 (15.19) T2 86.86 (15.72)	T0 63.61 (16.39)	T1 82.15 (17.52) T2 81.32 (17.80)	EG & CG: T1 Yes T2 No	EG: T0‐T1: 0.57 T0‐T2: 0.54 CG: T0‐T1:0.48 T0‐T2:0.46
Baek et al. (2019) (Non‐RCT‐two groups)	66: EG (*n* = 35); CG (*n* = 31)	*Instrument*: Self‐efficacy regarding SML instrument *Variables*: Self‐efficacy	16.61 (3.58)	21.14 (2.97)	17.25 (3.20)	17.70 (3.97)	EG & CG Yes	EG: 0.57 CG: 0.06
Parvizy (2020) (Non‐RCT‐two groups)	60	*Instrument*: The PNKAS self‐efficacy questionnaires *Variables*: Self‐efficacy	18.06 (No SD)	21.3 (No SD)	17.1 (No SD)	20.36 (No SD)	EG & CG: No	NCC
Sharour (2019) (Non‐RCT‐two groups)	70 EG (*n* = 35); CG *n* = 35)	*Instrument*: The GSE general self‐efficacy scale *Variables*: Self‐efficacy	23.2 (2.31)	35.50 (3.25)	22.8 (2.21)	24.25 (2.85)	EG & CG Yes	EG: 0.91 CG: 0.27
Derikx (2014) RCT	30 (*n* = 15); RNAs device A	*Instrument*: The Self‐efficacy in resuscitation measured by a visual analogue scale (VAS) *Variables*: ‐ RNs Self‐efficacy	RNAs: T0: 6.9 (1.9)	RNAs: T1: 6.1 (1.6) T2:5.7 (2.2)	NDR	NDR	NDR	RN: T0‐T1: −0.22 T0‐T2: −0.28
Iannuzzi (2019) RCT	14: EG 6; CG 8	*Instrument*: Family Nurse Practitioner Autism Self‐Efficacy Scale (FNPASE) *Variables*: Self‐efficacy	49.0 (6.7)	91.7 (11.0)	48.9 (11.8)	54.0 (8.7)	NDR	EG: 0.91 CG: 024
Larsen (2011) (Non‐RCT‐two groups)	39: EG (*n* = 14); CG (*n* = 25)	*Instrument*: Transcultural Self‐Efficacy Tool *Variables*: Cognitive SE Practical SE Affective SE	169.5 (34.9) 196 (36.0) 257.7 (21.2)	206.4 (31.0) 230.5 (37.3) 271.4 (25.3)	NDR	NDR	EG: Yes Yes Yes CG: NDR	EG: 0.49 0.43 0.28 CG: NCC
Moeini (2019) RCT	52	*Instrument*: Self‐efficacy of clinical performance questionnaire *Variables*: self‐efficacy	53.23 (10.5)	72.22 (10.50)	NDR	NDR	EG Yes CG No	EG: 0.69 CG: NCC
Gotwals (2018) (Non‐RCT‐two groups)	92: EG (*n* = 55); CG (*n* = 37)	*Instrument*: Health Promotion Counselling Self‐Efficacy scale (HPCSE) *Variables*: ‐ Nutrition knowledge self‐efficacy ‐ Nutrition counselling self‐efficacy	NDR NDR	NDR NDR	NDR NDR	NDR NDR	EG and CG: Yes ED and CG Yes	NCC
Phuangngoenmak (2019) RCT	60: EG 30; CG 30	*Instrument*: Perceived Self‐efficacy in DM Care questionnaire *Variables*: Self‐efficacy	T0: 57.96 (6.4)	T1: 71.60 (3.6) T2: 72.23 (3.2)	T0: 54.93 (11.2)	T1: 57.96 (6.4) T2: 57.96 (6.4)	EG & CG T0‐T1 Yes T0‐T2 Yes	EG: T0‐T1: 0.80 T0‐T2: 0.82 CG: T0‐T1:0.16 T0‐T2: 0.16
Topbaş et al. (2019) RCT	56: EG 28; CG 28	*Instrument*: General Self‐Efficacy Scale (GSES) *Variables*: Self‐efficacy	30.21 (6.34)	31.79 (5.39)	30.43 (4.57)	32.57 (6.94)	T0‐T1 EG YES EG & CG No	EG: T0‐T1: 0.13 CG: T0‐T1: 0.18
Mun and Hwang (2020) RCT	56: EG 28; CG 28	*Instrument*: Self‐efficacy scale for chemotherapy nursing scale *Variables*: Self‐efficacy	36.54 (3.28)	37.32 (3.35)	35.54 (3.53)	37.25 (2.59)	EG & CG T0‐T1 No	EG: T0‐T1: 0.12 CG T0‐T1: 0.27

*Note*: T0 time 0 (pre‐test), T1 time 1 (post‐test), T2 time 2 (delayed post‐test); T3 time 3 (delayed post‐test); T4 time 4 (delayed post‐test).

Abbreviations: CG, control group; EG, experimental group; M, mean; NA, not available; NCC, no calculation completed; NDR, no scale data for nursing students reported; RN, Registered Nurse; SD, standard deviation; SE, self‐efficacy.

## 4 EFFECTIVE SELF‐EFFICACY EDUCATION STRATEGIES

Various learning interventions targeting nurses' self‐efficacy were reported across the included studies (*n* = 47) (Appendix S3). The interventions were broadly categorized as online learning approaches, simulation learning approaches, traditional didactic approaches and blended learning approaches.

### 4.1 Online learning approaches

#### 4.1.1 Single modality

Four studies, 4/47 (8.5%) used a pre‐and post‐test design. They used online learning approaches using a range of multimedia systems, including videos, pictures, text, diagrams, and case scenarios lasting between 30 min and 3 months. All four studies reported a significant effect of training on self‐efficacy outcomes, with effect sizes *r* ranged from 0.28 to 0.63 (Chang et al., 2020; Curtis et al., 2016; Doucet & Rhéaume, 2020; Thompson, 2016) (see Appendix S3). Only one study, 1/4 (25%), assessed the impact of self‐efficacy in improving the transition of nursing students from education to clinical practice (Thompson, 2016). This study found that the self‐efficacy of undergraduate students improved as they transitioned from academic settings to professional practices when responding to bullying.

### 4.2 Simulation learning approaches

#### 4.2.1 Single‐modality simulation learning approaches

Four studies 4/47 (8.5%) used only simulations, and all of these studies reported positive effects on self‐efficacy 4/4 (100%). These studies assessed the impact of educational interventions on context‐specific self‐efficacy, such as on patient communication and physical care (Dunn et al., 2014); respiratory problems and cardiac scenarios (Saied, 2017); feeding tube insertion (Bourgault et al., 2019); counsel about nutritional health promotion and disease prevention (Gotwals, 2018). The majority of these interventions involved specific skills required for assessing and caring for patients.

#### 4.2.2 Multimodality simulation learning approaches

Less than a quarter 10/47 (21.3%) of the studies used simulation in combination with other educational approaches. Results showed that post‐test self‐efficacy was higher than pre‐test self‐efficacy in the nine studies. Among the intervention groups, 60% used lectures (*n* = 6), and 10% used low‐fidelity manikins (*n* = 1). Simulation sessions were based on communication with a patient experiencing mental health issues (Kameg et al., 2010), maternity scenarios (Said et al., 2021), managing multiple simulation patients (Franklin et al., 2015), team training (Leithead et al., 2019), examining the graduate nursing students self‐efficacy of counselling abilities among individuals with mental illness (Schwindt et al., 2019), septic shock and chemotherapy reaction management (Sharour, 2019), faculty self‐efficacy (Crocetti, 2014) and effects of a visiting nursing simulation on nursing students' self‐efficacy (Hwang & Kim, 2020).

### 4.3 Traditional didactic learning approaches

Of the 47 studies, 44.7% (*n* = 21) were didactic‐based interventions; 20 of these studies (20/21, 95.2%) showed statistically significant higher means for the post‐test vs. the pre‐test groups with a range of different teaching strategies/resources for improving self‐efficacy such as in a sexual education programme (SEP) context, posters and graphics promoting self‐efficacy (Baek et al., 2019), and lectures and didactic presentations (Kim et al., 2018). In addition, palliative care training that includes lectures and workshops has been shown to improve nurses' perceived self‐efficacy (Dehghani et al., 2020), and the training in pain management, which incorporates lectures, group discussions, and case studies was found to be effective in improving paediatric nurses' self‐efficacy (Parvizy et al., 2020) (see Appendix S3).

### 4.4 Blended learning interventions (Didactic methods, workshops, role‐play, videos, reflective practice)

Ten studies 10/47 (21.3%) used a blended approach in providing training interventions to registered nurses and nursing students, and 100% of these studies were effective in achieving their primary outcomes (Appendix S3). The duration ranged from 20 min to 5 weeks (Franklin et al., 2015; Said et al., 2021). Different teaching modalities were used through a combination of lectures, small groups (debriefings), videos, team‐based learning, computer‐based learning, individual projects and problem‐based learning that proved to be effective in different studies (Crocetti, 2014; Franklin et al., 2015; Hwang & Kim, 2020; Kameg et al., 2010; Leithead et al., 2019; Phuangngoenmak et al., 2019; Schwindt et al., 2019; Sharour, 2019; Said et al., 2021; Walker et al., 2014; Topaş et al., 2019).

In one study, a blended approach was used to measure the long‐term effects of a diabetes education intervention (3 weeks of classroom training followed by five eLearning modules) (Phuangngoenmak et al., 2019). The results showed a significant increase in self‐efficacy in the experimental group (EG) compared with the control group (CG) from pre‐test to post‐test at 4 weeks; pretest (EG, (m = 71.60 (SD = 3.6))) V CG (m = 57.96 (SD = 6.4), *p* = 0.007) and at 8 weeks (EG, (m = 72.23 (SD = 3.2))) V CG (m = 57.96 (SD = 6.4), *p* = 0.002).

### 4.5 Education programmes characteristics leading to increased self‐efficacy

Eighteen studies included a guided reflection/debriefing component and positively affected participants' self‐efficacy. Debriefing sessions were shown to help participants learn more effectively (Schwindt et al., 2019). Interventions incorporating debriefing included group supervision and peer learning (Crocetti, 2014; Kameg et al., 2010; Leithead et al., 2019; Rambod et al., 2018; Sharour, 2019); clinical scenarios (Crocetti, 2014; Hwang & Kim, 2020; Saied, 2017); role‐play (Alfes, 2015; Kool et al., 2014); classroom training combined with e‐learning (Phuangngoenmak et al., 2019); focus groups (Hwang & Kim, 2020; Said et al., 2021; Schwindt et al., 2019).

### 4.6 Self‐efficacy in the context of the transition of nursing students from an educational programme into clinical practice

Although some of the included studies were longitudinal and repeated self‐efficacy scores after a semester or a year, there was limited evidence on self‐efficacy in the context of transitioning nursing students from an educational programme into clinical practice. Of 47 studies, one study used an online educational module to support the development of self‐efficacy relating to dealing with bullying behaviour; the study sample included undergraduate nursing students transitioning from the academic setting to professional practice (EG M = 32.1, SD = 3.7, CG M = 29.6, SD = 3.6; *p* < 0.001) (Thompson, 2016).

## 5 DISCUSSION

In this systematic review, evidence from interventions that promote self‐efficacy was identified, critically evaluated and synthesized, along with examining the role self‐efficacy plays in the transition from clinical to academic practice. 47 papers were included, and over two‐thirds (35/47) of interventional studies demonstrated improvements in self‐efficacy levels. Our results corroborated previous studies reporting the effects of such programmes on nurses' self‐efficacy (Ardakani et al., 2019; George et al., 2017), these results confirm the effectiveness of nursing education programmes. A variety of research designs, sampling strategies, sample sizes, interventions, teaching and learning approaches and programme durations impact conclusions regarding how to increase self‐efficacy.

Even though some papers did not present comparative statistics (e.g. Alfes, 2015; Bourgault et al., 2019; Crocetti, 2014; Hagemeier et al., 2014; Moore et al., 2019; Said et al., 2021), a significant change in learners' self‐efficacy was reported in 35 studies. This supports the idea that self‐efficacy can be developed through targeted education programmes. It is consistent with a previous systematic review (including 19 articles) carried out by McCutcheon et al. (2015), which showed that online interventions significantly enhanced self‐efficacy in performing nursing skills compared to face‐to‐face learning. Also, according to Mata et al. (2021), a systematic review of eight randomized controlled trials (RCTs) and quasi‐experimental studies concluded that communication skills training improves nurses' performance and self‐efficacy. Similarly, Patricio‐Gamboa et al. (2021), in a systematic review (*n* = 26 included papers), also reported that perceived self‐efficacy and academic performance were directly related in the broader educational context.

This review described five categories of interventions (single‐modality online learning, single‐modality simulation‐based learning, multimodality simulation, didactic teaching and learning methods and clinical training). Four studies have shown that online learning is more effective than face‐to‐face learning for acquiring self‐efficacy. However, combining online and other practice‐based approaches may provide a more comprehensive blended approach. (Doucet & Rhéaume, 2020; Iannuzzi et al., 2019). Nevertheless, it was difficult to make a conclusive judgement due to the lack of evidence available. The limited evidence suggests a need to research how to improve nursing self‐efficacy through online learning approaches.

Within the reviewed literature, face‐to‐face approaches positively impacted self‐efficacy in a larger number of included studies. Face‐to‐face interventions tended to have a larger effect size than other methods. With reference to simulation‐based learning, the results suggest that multimodality approaches, such as simulations combined with other instructional methods, are more useful in developing self‐efficacy for those who are less skilled, while a single simulation‐based approach may provide greater benefits for those with more clinical experience in terms of developing self‐efficacy and are moving from novice to expert (Benner, 1984). As a result, they are developing specialist skills associated with greater complexity.

There were variations in results between studies, and this might be due to differing operationalizations of self‐efficacy and tools used in measuring self‐efficacy, reflecting different aspects of self‐efficacy. In this review, 10 studies used the general self‐efficacy scale GSES (*n* = 10) (see Appendix S4) to evaluate the effects of educational interventions, while the remaining studies used a large variety of tools. Measuring a nurse's self‐efficacy can be difficult, and comparison across studies is problematic due to variation in tools (37 tools sourced); and the focus on task/domain‐specific self‐efficacy (such as interprofessional self‐efficacy, mental health nursing self‐efficacy, and transcultural self‐efficacy (*n* = 37 studies) in contrast to general self‐efficacy (*n* = 10 studies)). Five of the included studies provided validity and reliability data on the self‐efficacy tool (Alfes, 2015; Ataee et al., 2019; Daglar, 2018; Rambod et al., 2018; Topbaş et al., 2019). Other authors have commented on the need for researchers to consider the psychometric properties of self‐administered instruments to ensure consistency in measurement and the highest quality of measurement (Hwang & Kim, 2020; Saxton,&nbsp;^2012^).

Complex educational interventions have several interacting components. However, evidence on the impact of individual programme components is limited. For example, a supplementary effect of reflective practices or debriefing sessions may have been responsible for the significant results achieved by simulated intervention groups. Furthermore, human interaction and feedback may facilitate educational interventions in enhancing self‐efficacy (Hwang & Kim, 2020; Phuangngoenmak et al., 2019).

This review shows that nursing education interventions affect nurses' and nursing students' self‐efficacy. The evidence is primarily non‐randomized, based upon small sample sizes, with data collected over relatively short timeframes which limits our ability to definitively link changes in self‐efficacy to certain programme components. While authors sought to synthesize details of statistically effective interventions (Appendix S3); without meta‐analyses and more longitudinal data, author conclusions have to be tentative. This review is important because it is one of the only reviews to focus on this topic area with an interventional focus. Self‐efficacy in the context of nursing education is important because if students believe in their capabilities, they would use their maximal efforts in different situations. Moreover, when the self‐efficacy of student nurses is developed, their achievement motivation is also improved. This review provides us with several take‐home messages for nurse educators: some teaching and learning strategies appear more regularly in successful interventions, that is flipped classroom approaches, simulation, debriefing, and role‐play; multimodal approaches seem to be more effective than single‐mode educational interventions; researchers should provide more details of intervention components in line with recommendations.

## 6 STRENGTHS AND LIMITATIONS

This review contributes to the limited evidence base regarding educational interventions that build self‐efficacy in nursing education (McCutcheon et al., 2015). An overview of self‐efficacy findings from the last decade is presented in the review. This summary intends to highlight areas in need of further research, which may assist in planning future studies.

However, this review is limited by a lack of rigorous and high‐level evidence about the types of education programmes/interventions that cultivate self‐efficacy most effectively. Additionally, based on our findings, studies included in the review lacked theoretical underpinnings. Over 60% of included studies lacked theoretical underpinnings, which is essential to developing and planning interventional studies (Iannuzzi et al., 2019). Thirdly, MMAT scores of 50 or below were found in nearly 60% of the studies. Fourth, the results provide little insight into the long‐term relationship between self‐efficacy and educational programmes. Only one study included longitudinal follow‐up on participants. Further research is needed to explore how self‐efficacy facilitates the transition from education to clinical practice for nursing students. The fourth limitation concerns the limited number of RCTs sourced; we found only eight eligible trials. Therefore, the conclusions of the review should be interpreted with caution. Additionally, the research team determined the search strategy without librarian support, including identifying databases and running the searches. Also, for this review, the absence of reported effect sizes for most of the included studies may indicate a weakness in the body of literature. Finally, meta‐analyses were not conducted due to the heterogeneity of designs, data collection instruments, education programmes, sample sizes, data collection settings and outcome measures.

## 7 CONCLUSION

The review included a large body of literature (*n* = 47 studies), with the majority of empirical research in this area occurring in the United States (*n* = 21) and very little in Europe (*n* = 2). Limited higher‐level evidence (e.g. randomized trials) was sourced, with the majority of studies being single groups, with no control group. There was a limited application of theory underpinning interventional studies. A large variety of tools were described, and most studies used general self‐efficacy and researcher‐designed scales.

This review demonstrates that nursing education programmes can develop and nurture self‐efficacy. Some authors have proposed that self‐efficacy has a mediating role in terms of bridging the gap between theoretical and practical knowledge, behaviours and clinical practice.

The majority of interventional studies statistically impacted self‐efficacy; however, we cannot definitively conclude which educational interventions optimize self‐efficacy. We can tentatively hypothesize that multimodal interventions are better than single‐intervention studies and that simulation appears to support the development of self‐efficacy in targeted skills/areas. Self‐efficacy is an important variable in supporting nurses in making key transitions. However, limited empirical understanding of its role in the transition from student to practitioner was sourced, and longitudinal studies were lacking.

## AUTHOR CONTRIBUTIONS

MA, NW, RC, BR and JH contributed to study design, data collection, data analysis and manuscript writing and supervised the study. All authors agreed on the final version.

## FUNDING INFORMATION

There were no public, commercial or nonprofit organizations that funded this study.

## CONFLICT OF INTEREST STATEMENT

The authors report no conflicts of interest.

## ETHICS STATEMENT

There was no ethical approval needed since this is a systematic review of primary studies.

## Supporting information


Appendix S1

Appendix S2

Appendix S3

Appendix S4

## Data Availability

Data openly available in a public repository that issues datasets with DOIs
